# Physical Activity-Related Language and Psychosocial Themes in a Psychological AI-Training Q&A Corpus: An Exploratory BERTopic Analysis

**DOI:** 10.3390/healthcare14162547

**Published:** 2026-08-14

**Authors:** Yuze Zhang, Yinghai Liu, Yang Wang, Yanlan Guo

**Affiliations:** 1 College of Physical Education, Shanxi University, Taiyuan 030006, China; zhangyuze@sxu.edu.cn (Y.Z.); wangyang13@sxu.edu.cn (Y.W.); 2 College of Physical Education, North University of China, Taiyuan 030051, China; 20250073@nuc.edu.cn

**Keywords:** psychological support text, physical activity-related language, physical education, BERTopic, student–AI interaction corpus, topic modelling, emotional regulation, self-determination theory, exploratory text mining

## Abstract

**Background:** Q&A corpora generated through university student–AI mental health support tools may reveal how physical activity (PA) and psychosocial themes are represented in support-oriented text. However, the absence of individual-level demographic metadata and the pooling of prompt and response fields limit attribution of any expression to a particular speaker, and the corpus describes a specific student population rather than a general or clinical one. **Objective:** This exploratory study described PA-, sport-, physical education (PE)-, body-, lifestyle-, and emotion-related patterns in a large corpus of university student–AI mental health exchanges collected through an institutional counselling platform. **Methods:** This study analysed 209,715 paired prompt–response records as combined exchange-level units using a BERTopic-based computational text-mining workflow. The full corpus was used for the main 18-topic model and overlapping dictionary analyses. After secondary data-quality filtering, 178,062 eligible exchanges formed the sampling frame from which a systematic sample of 10,000 exchanges was drawn for a separate complementary BERTopic and scenario-mapping analysis. The workflow used Qdrant/bge-small-zh-v1.5 embeddings, NFKC normalisation, an archived stop-word list, UMAP (n_neighbors = 15, n_components = 5, min_dist = 0.0, cosine metric, seed = 42), HDBSCAN (min_cluster_size = 300, min_samples = 10, Euclidean metric, EOM), c-TF-IDF topic representations, overlapping dictionary screens, and stability testing across seeds 42, 52, and 62. **Results:** A student/school/family-context lexical screen matched 83,215 exchanges (39.68%), and a broad PA/body/lifestyle screen matched 82,464 exchanges (39.32%). These overlapping indicators describe topical co-occurrence and do not establish PA behaviour or which party to the exchange produced a given term. Eighteen corpus-level themes were retained. In the 10,000-exchange analysis, 13.11% of exchanges matched a narrow movement-related expression screen, with the highest within-topic rate in the sample topic labelled emotional outburst and relaxation regulation (51.09%). **Conclusions:** The findings describe exchange-level lexical and topic patterns in student–AI interactions rather than actual PA behaviour, intervention delivery, clinical efficacy, or population prevalence, and they do not identify which party introduced the language. The mapping to autonomy, competence, relatedness, and emotional regulation is a post hoc interpretive lens, offered as a hypothesis to inform future, prospectively validated design work in PE and digital mental health support rather than as a demonstrated result.

## 1. Introduction

Mental health concerns among adult university students are widely recognised as important challenges for education, social participation, and long-term well-being. Emotional distress, loneliness, academic pressure, family conflict, and low self-worth may affect engagement in school, relationships, and healthy lifestyle development [1,2,3,4]. Within the 2030 Agenda, education is increasingly understood as extending beyond knowledge acquisition to include social–emotional well-being, equity, and opportunities for healthy living [5,6]. Adult university students are the population addressed in this discussion, although psychological-support materials may also include adult, family, and general mental health contexts [7,8,9].

Physical activity (PA), sport, and physical education (PE) have been associated with several psychosocial outcomes, including mood, sleep, self-efficacy, and social interaction [10,11,12,13]. However, their relationship with mental health is neither uniform nor automatically beneficial. Movement and sport may provide enjoyment, social connection, and emotional relief, but they may also be discussed in relation to body dissatisfaction, peer comparison, perceived failure, social anxiety, or performance pressure [14,15,16]. Understanding the context in which movement-related language appears is therefore important when considering its possible role in psychological and educational support.

Most previous research on PA and mental health has relied on questionnaires, population surveys, or intervention studies. Large textual datasets offer a complementary opportunity to examine how PA-, sport-, body-, lifestyle-, and emotion-related language occurs within support-oriented Q&A materials. Nevertheless, limited research has examined these patterns in large psychological AI-training corpora. Such corpora also present important interpretive difficulties. When record-level provenance and demographic metadata are unavailable, the material cannot be assumed to represent authentic consultations or the language of a defined population. Moreover, when prompt and response fields are combined, it is impossible to determine which field introduced a particular expression.

The present exploratory study examines more than 200,000 paired psychological AI-training Q&A records as exchange-level textual units. In this study, an “AI-training Q&A record” refers to a prompt paired with an associated response and compiled for model-training purposes. The term does not indicate that the record represents a verified clinical encounter or an output produced by an AI system evaluated in this study. The study aims to describe corpus-level topic patterns and lexical co-occurrences involving PA, sport, PE, body, lifestyle, emotional, and student/school/family-context expressions. Student/school/family-context terms are treated only as lexical indicators and do not verify the age or identity of any speaker. Accordingly, the study does not infer individual behaviour, motivation, diagnosis, clinical need, or population prevalence, nor does it develop or validate a clinical AI system.

## 2. Literature Review and Analytical Framework

### 2.1. Physical Activity, Mental Health and Education Contexts

Physical education and health are increasingly discussed as educational fields that connect movement, well-being, social inclusion, and the development of life skills [17,18,19]. Research has reported consistent associations between PA and several mental health outcomes, although the direction, strength, and mechanisms of these relationships are complex. Proposed pathways include self-efficacy, sleep, social support, enjoyment, and perceived competence [20,21,22].

Within research involving adult university students, an important question is not only whether adult university students participate in PA, but also how their movement experiences are perceived and socially organised [23,24]. Sport and PE may provide enjoyment, belonging, and opportunities for emotional regulation, but they may also be associated with evaluation, body-image concerns, perceived failure, exclusion, or performance pressure [25,26,27,28]. From an SDT-informed perspective, the educational value of movement experiences may be enhanced when they support autonomy, competence, and relatedness, while also providing appropriate opportunities for emotional regulation [29,30,31,32].

### 2.2. Motivation and Psychosocial Needs in Physical Education

Self-determination theory provides a potentially useful interpretive framework for examining movement-related and psychosocial patterns in paired Q&A exchanges [33,34,35,36]. Autonomy refers to experiencing one’s actions as self-endorsed; competence concerns feeling capable and effective; and relatedness refers to feeling accepted, connected, and supported by others [37,38,39,40,41]. In educational and PA settings, frustration of these needs may be associated with avoidance, reduced engagement, perceived pressure, or negative movement experiences [42,43,44].

Movement experiences may also be shaped by peer evaluation, family expectations, body-image concerns, teacher feedback, and the wider school environment [45,46,47,48]. These factors may influence whether PA or sport is experienced as supportive, neutral, or burdensome [49,50,51,52,53]. In the present study, however, autonomy, competence, and relatedness were not directly measured or coded using a validated SDT instrument. SDT was used only as a post hoc interpretive lens for considering whether corpus-level textual patterns might be relevant to these constructs. Emotional regulation was treated as a related but conceptually distinct interpretive domain. These theoretical correspondences are therefore hypothesis-generating rather than confirmed psychological classifications.

### 2.3. BERTopic and Psychological Q&A Text Analysis

Topic modelling can assist in identifying recurring semantic patterns in large textual datasets [54,55,56,57]. BERTopic combines document embeddings, dimensionality reduction, clustering, and class-based TF-IDF topic representation to group semantically similar texts and generate interpretable topic terms [58,59,60,61,62]. Compared with simple keyword frequencies, this approach can identify semantically related expressions that use different surface vocabulary.

In the present study, BERTopic provided one primary model-generated topic assignment for each paired exchange. These assignments were analytically distinct from the overlapping dictionary screens used to identify PA-, sport-, PE-, body-, lifestyle-, and emotion-related expressions. Automatically generated topic terms were subsequently reviewed through post hoc team interpretation and consolidated into descriptive corpus-level labels. The sample-based scenario screens were also multi-label and separate from the BERTopic assignments.

To evaluate the reproducibility of the post hoc interpretive layer, all 18 topic labels and SDT-related mappings were subjected to a retrospective independent re-evaluation using a coding manual frozen before the two rating records were compared. The audit assessed topic-label adequacy and the presence of autonomy, competence, relatedness, and emotional regulation; it did not treat these mappings as validated psychological measurements. The procedure and results are reported in Section 3.6 and Section 4.2.

## 3. Materials and Methods

### 3.1. Study Design and Analytical Scope

This study employed an exploratory computational text-mining design to examine physical activity-related and psychosocial language in paired university student–AI mental health Q&A exchanges collected through a university counselling platform. The study involved the secondary analysis of existing textual records and did not include direct participant recruitment, clinical assessment, or the implementation or evaluation of a physical activity, psychological, or clinical intervention. Each record consisted of a mental health-related question or prompt submitted by a university student and the corresponding response generated by a generative-AI system integrated into a student mental health counselling platform. The student prompt and AI-generated response were concatenated before corpus-level modelling. The unit of analysis was therefore the complete student–AI exchange rather than an individual student statement or AI-generated response.

Prompt and AI-generated responses were combined before analysis; therefore, the analytical unit was the complete prompt–response exchange rather than an individual prompt, response, or speaker statement. As a result, specific words, topics, concerns, or recommendations could not be attributed to either the student prompt or the AI-generated response. All findings are therefore reported as exchange-level textual patterns and should not be interpreted as reflecting the spontaneous language, behaviour, needs, or recommendations of a particular speaker. Moreover, the analysis does not establish students’ physical activity behaviour, psychological diagnoses, treatment needs, intervention exposure, or the clinical quality, safety, or appropriateness of the AI-generated responses.

**Figure 1:**
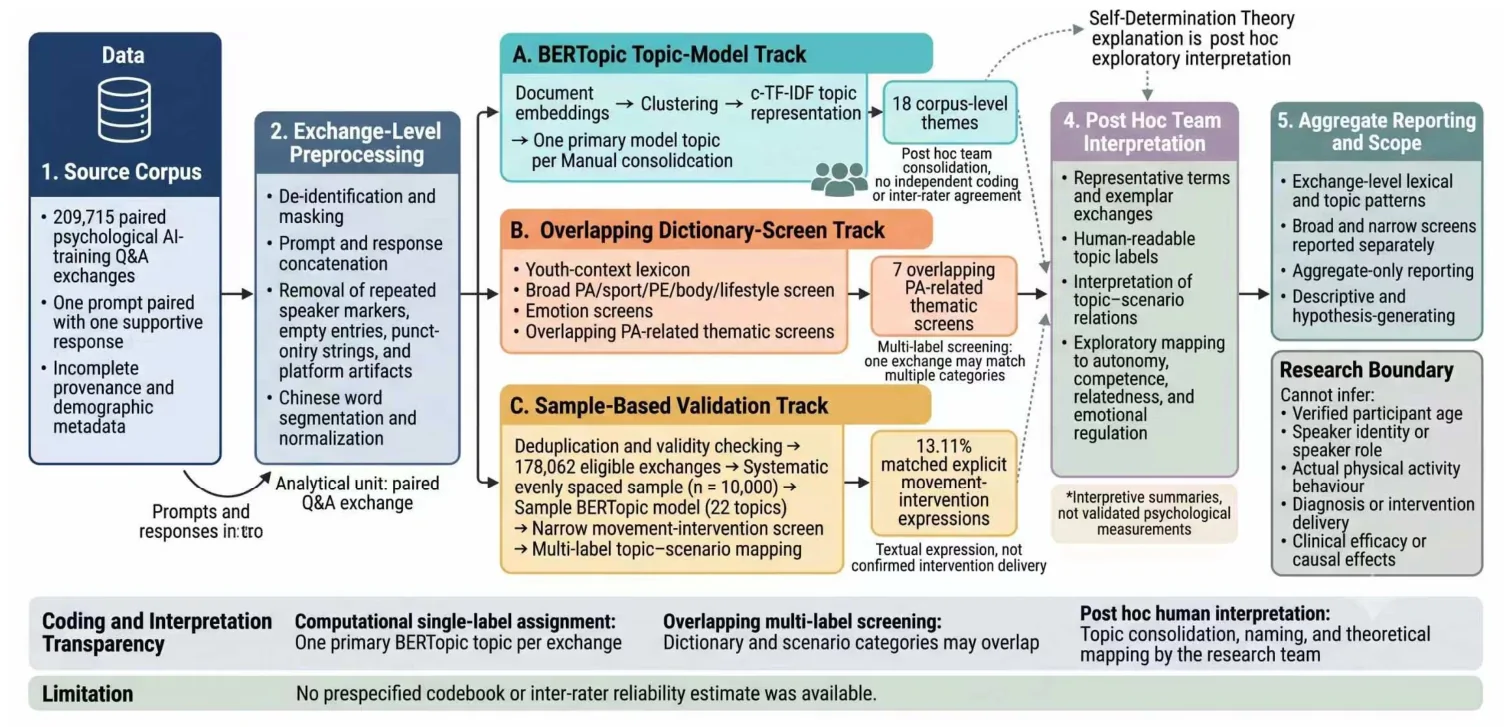
Analytical and coding workflow distinguishing model assignment, overlapping lexical screens, post hoc interpretation, sample validation, and the retrospective independent topic-label and SDT-mapping reliability audit.

The analytical framework comprised five components as shown in Figure 1: (1) corpus-level BERTopic (v0.17.4) modelling; (2) overlapping dictionary-based lexical screening; (3) post hoc interpretation by a five-member multidisciplinary team spanning computer science, health sciences, psychology, and sports science, with disagreements resolved by consensus; (4) a complementary topic and scenario analysis based on a systematic sample of 10,000 exchanges drawn from the filtered 178,062-exchange sampling frame; (5) a retrospective independent human reassessment of all 18 topic labels and SDT-related mappings. BERTopic assignments, dictionary-screen matches, scenario-screen matches, and theoretical interpretations were treated as separate analytical layers throughout. The corpus-level model analysed all 209,715 exchanges and retained 18 topics, whereas a separate model fitted to the systematic 10,000-exchange sample retained 22 topics for the complementary scenario analysis.

### 3.2. Data Source and Study Population

The source corpus was provided by Shanxi University and collected through a student mental health counselling platform into which a generative-AI response function had been integrated. The corpus included interactions involving university students enrolled across universities throughout Shanxi Province, China, between 2024 and 2026. Each record contained a mental health-related question or prompt submitted by a university student and the corresponding response generated by the platform’s generative-AI system; the records therefore represent student–AI interactions rather than counsellor–patient consultations. All records were confirmed by the research team to be authentic student–AI interactions; the corpus contains no test records, demonstration cases, manually curated examples, translated or rewritten content, or synthetic training cases.

The Shanxi University Ethics Committee approved the secondary analysis of the de-identified dataset (protocol SXULL2026092; 10 April 2026) and waived individual informed consent because the study involved retrospective analysis of existing de-identified textual records, no participant contact or re-identification, no reproduction of raw passages, and aggregate-only reporting. Shanxi University formally authorised use of the records under its institutional data-governance procedures.

The source corpus contained 209,715 paired exchanges. This number represents Q&A records rather than unique students; one student could contribute multiple exchanges and stable anonymous student identifiers were unavailable. The study population consisted exclusively of enrolled university students aged 18 years or older. All study-population descriptions now consistently refer to adults.

Authorised research team members extracted and de-identified the records before analysis, removing or masking direct personal identifiers including names, contact details, account identifiers, and other identifying fields where present; no attempt was made to identify or re-identify individual students. The de-identified corpus was stored on secure servers maintained by Shanxi University, with access restricted to authorised research team members through institutionally managed accounts. Because the corpus nonetheless contains sensitive mental health content, including self-harm- and suicide-related language, no raw passage is reproduced in this manuscript and results are reported only in aggregate; residual deductive-disclosure risk arising from unusual combinations of narrative detail is the reason the raw corpus is not made publicly available.

### 3.3. Text Preprocessing and BERTopic Pipeline

Source integrity was assessed directly against the original workbook, which contained 209,715 paired prompt–response exchanges. The main corpus-level BERTopic analysis was conducted using all 209,715 combined exchanges. A secondary data-quality filtering procedure was then applied to create a sampling frame for the complementary sample-based analysis. This procedure retained 178,062 eligible exchanges, from which a systematic sample of 10,000 exchanges was selected. The filtered frame was not used to generate the 18 corpus-level topics reported in the following text. The secondary filtering procedure excluded 31,653 records that did not satisfy the sample-analysis completeness, formatting, and eligibility rules, leaving 178,062 exchanges eligible for systematic sample selection.

The frozen pipeline applies NFKC normalisation, removes specified speaker markers, collapses whitespace, and joins the normalised prompt and response with a newline. Embeddings are generated using Qdrant/bge-small-zh-v1.5 at immutable revision, with CLS pooling, L2 normalisation, right truncation at 512 tokens, and a batch size of 16. UMAP uses n_neighbors = 15, n_components = 5, min_dist = 0.0, cosine distance, low_memory = True, n_jobs = 1, and seed 42. HDBSCAN uses min_cluster_size = 300, min_samples = 10, Euclidean distance, EOM cluster selection, and prediction_data = True. Topic representations use jieba 0.42.1 precise segmentation, the archived 181-term stop-word resource, unigram–bigram features, min_df = 5, max_df = 0.98, and c-TF-IDF. Initial clusters are reduced to 18 topics with BERTopic.reduce_topics; outliers remain coded −1 and are reported separately.

Stability is evaluated across UMAP seeds 42, 52, and 62 using adjusted Rand index and normalised mutual information for all documents and jointly non-outlier documents. Topic quality is summarised using document-level NPMI for the top 10 terms and topic diversity.

### 3.4. Physical Activity and Emotion Dictionaries

The complete dictionary resource prespecifies the broad PA/body/lifestyle screen, a narrower explicit-movement screen, the student/school/family-context screen, eight emotion screens, and seven PA-related thematic screens. Matching is performed after NFKC normalisation by case-insensitive Unicode substring search. An exchange is counted once per category; categories may overlap, and no automatic negation inversion is applied. Generic body, weight, diet, sleep, breathing, and training expressions are included only in the broad or secondary screens and are excluded from the narrow movement definition. Dictionary matches indicate lexical co-occurrence rather than behaviour, diagnosis, speaker intent, or intervention delivery.

### 3.5. Complementary Sample-Based Topic and Scenario Analysis

For the complementary analysis, a systematic sample of 10,000 exchanges was drawn from the filtered sampling frame of 178,062 exchanges. The sampling interval was 17.8062; using seed 9986 and a one-based starting position of 12, record i was selected at floor [12 + (i − 1) × 17.8062]. All 10,000 selected positions were unique. A separate BERTopic model was fitted to the sampled exchanges and retained 22 sample-level topics. This model was independent of the corpus-level model that generated the 18 topics reported in Section 4.2.

### 3.6. Independent Topic-Label and SDT-Mapping Re-Evaluation

All 18 retained topics were the units of reassessment. The coding manual was frozen for this audit before the two rating records were compared; it was not a preregistration of the original BERTopic analysis or the original team interpretation. For each topic, the coders reviewed the proposed descriptive label, representative terms, inclusion criterion, and exclusion boundary. Topic-label adequacy was classified as accept or minor revision. Autonomy, competence, relatedness, and emotional regulation were coded separately as present (1) or absent (0) using theory-based operational rules. Emotional regulation was treated as related to, but conceptually distinct from, SDT’s three basic psychological needs. Each coder then selected one primary interpretive domain from the domains coded present, or not mapped if no domain met the criterion.

Two human coders completed separate records for all 18 topics and did not see one another’s decisions or notes before both records were locked. Raw percentage agreement and unweighted Cohen’s kappa were calculated before any discussion for topic-label adequacy, the primary interpretive domain, all 72 binary domain decisions (18 topics multiplied by four domains), and each domain separately. Because the four binary decisions were clustered within topic and the number of topics was small, 95% confidence intervals were obtained using a topic-cluster percentile bootstrap (10,000 replicates; seed 20260802). Disagreements were adjudicated only after reliability estimation; the original ratings were retained unchanged, and each final decision and rationale was logged. The coding manual, separate coder records, adjudication log, reliability summary, and executable script are included in the Supplementary Materials.

### 3.7. Research Questions

RQ1: What major topics are identified in the paired psychological AI-training Q&A exchanges using BERTopic?

RQ2: How frequently do broad PA-, sport-, PE-, body-, and lifestyle-related textual screens occur in the corpus?

RQ3: Which overlapping PA-related and psychosocial textual patterns co-occur at the exchange level?

RQ4: What cautious, hypothesis-generating design implications emerge from the sample-based topic–scenario mapping?

## 4. Results

### 4.1. Corpus Overview

Table 1 summarises the corpus and analytical scope. Among 209,715 paired exchanges, 83,215 (39.68%) matched the student/school/family-context lexicon and 82,464 (39.32%) matched the broad PA/body/lifestyle screen. This lexicon identifies topical references to school, family, or student life within an exchange; because the corpus population is already established as enrolled adult university students, the screen is used descriptively rather than as an age-verification tool. These overlapping dictionary indicators are descriptive and do not establish actual PA participation or whether a term appeared in the prompt or the response. The overlap was 35,141 exchanges (16.76% of the corpus; 42.61% of broad PA-screen-positive exchanges). The 209,715-exchange corpus was the denominator for the corpus-level BERTopic and overlapping dictionary-based analyses reported in Section 4.1, Section 4.2, Section 4.3, Section 4.4 and Section 4.5. The separate 178,062-exchange figure represents the filtered sampling frame used solely to select the complementary 10,000-exchange sample analysed in Section 4.6 and Section 4.7. It was not an alternative denominator for the corpus-level findings.

### 4.2. BERTopic-Derived Exchange Themes

**Figure 2:**
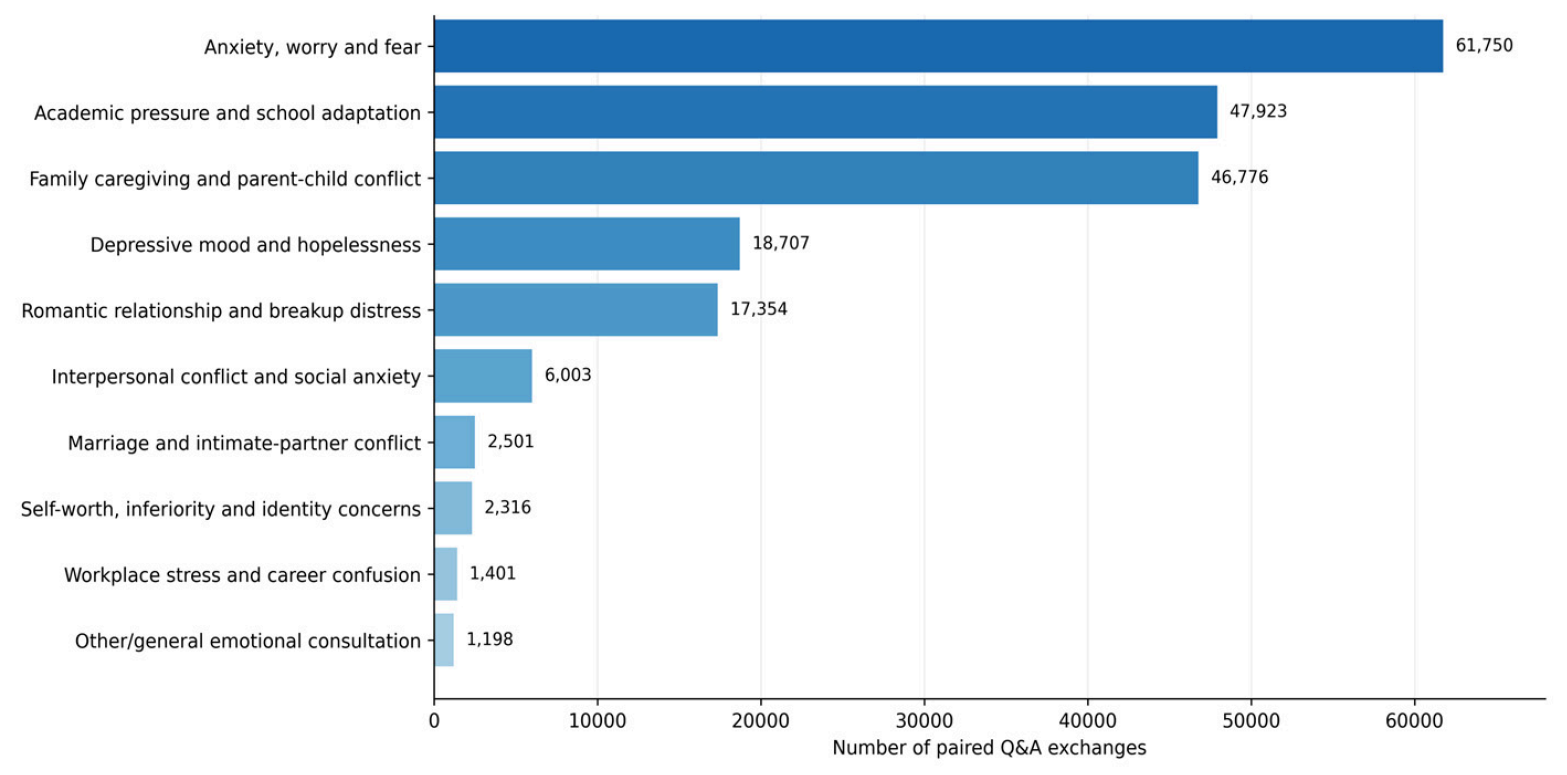
Ten largest corpus-level topics by number of paired Q&A exchanges.

Eighteen corpus-level topics were retained after modelling and post hoc consolidation; Table 2 reports the 10 largest. Each exchange contributed to one primary corpus-level topic, while the PA screen was an overlapping dictionary indicator. Figure 2 shows that anxiety, worry, and fear were the largest topic (61,750 exchanges; 29.44%), followed by academic pressure and school adaptation (47,923; 22.85%) and family caregiving and parent–child conflict (46,776; 22.30%). Within-topic PA-screen rates ranged from 49.47% for academic pressure and school adaptation to 0.75% for other/general emotional consultation. These rates describe lexical co-occurrence in pooled exchanges and do not identify the speaker or establish a behavioural or causal relation.

In the independent pre-adjudication reassessment, topic-label adequacy decisions agreed for 16 of 18 topics (88.9%; Cohen’s kappa = 0.750, topic-cluster bootstrap 95% CI [0.341, 1.000]). Primary-domain decisions agreed for 15 of 18 topics (83.3%; kappa = 0.764, 95% CI [0.505, 1.000]). Across the 72 binary domain decisions, agreement was 90.3% (65/72; kappa = 0.806, 95% CI [0.696, 0.917]). Domain-specific agreement and kappa values were 83.3% and 0.675 for autonomy, 94.4% and 0.886 for competence, 100.0% and 1.000 for relatedness, and 83.3% and 0.658 for emotional regulation. All disagreements were resolved only after these statistics had been calculated, with the original ratings preserved. These coefficients support the reproducibility of the interpretive audit but do not establish construct validity. The corpus-level BERTopic model was fitted to all 209,715 combined exchanges. Accordingly, the assigned-exchange counts and percentages reported in Table 2 use 209,715 as their denominator.

### 4.3. Physical Activity-Related Motivational and Psychosocial Themes

**Figure 3:**
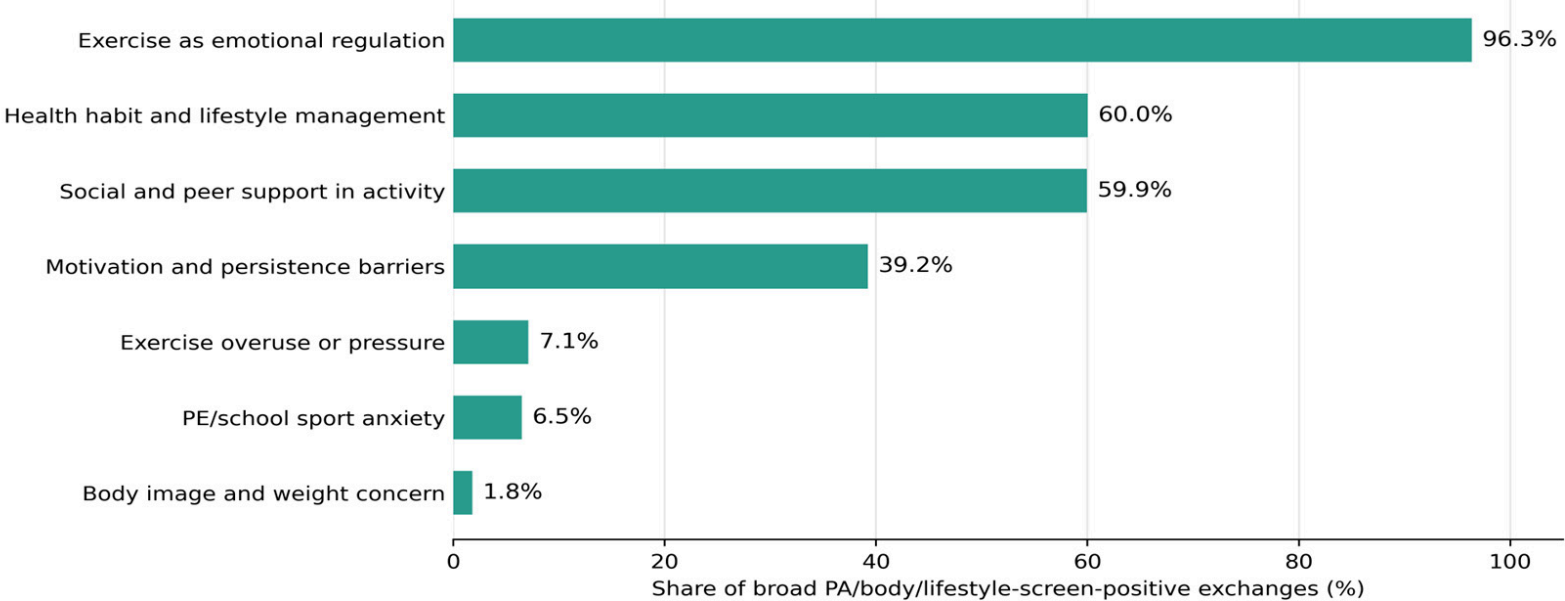
Distribution of overlapping physical activity-related thematic screens within the broad PA/body/lifestyle-screen-positive subset (n = 82,464).

Seven overlapping PA-related thematic screens were identified (Table 3, Figure 3). Co-occurrence of movement-related and emotional-regulation expressions matched 79,452 exchanges, equivalent to 37.89% of the full corpus and 96.35% of broad PA-screen-positive exchanges. This high percentage reflects nested, multi-label dictionary rules applied to combined prompt–response text; it does not mean that 96.35% of participants used exercise for regulation. Health habit/lifestyle management (60.03% of broad PA-screen-positive exchanges), social/peer support in activity (59.94%), and motivation/persistence barriers (39.22%) also overlapped substantially. Less frequent screens included exercise overuse/pressure (7.09%), PE/school sport anxiety (6.49%), and body image/weight concern (1.79%).

### 4.4. Emotional Profiles of the Corpus

**Figure 4:**
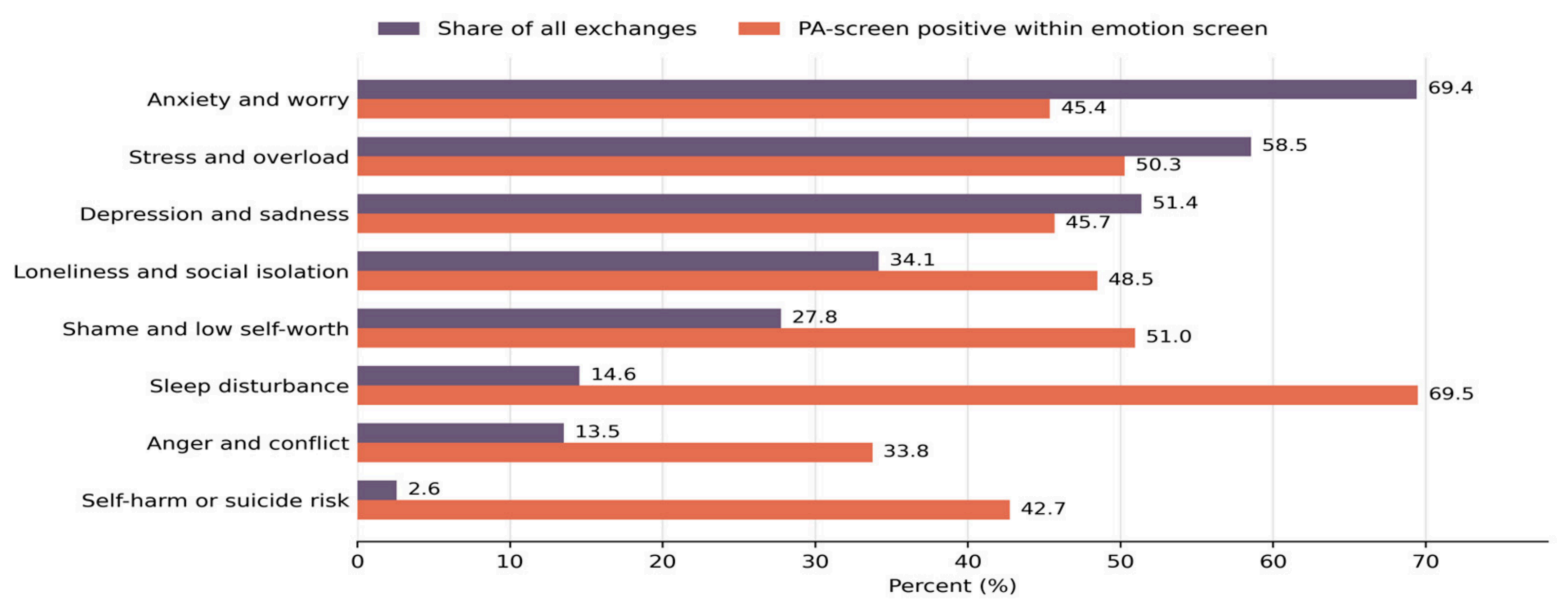
Emotion-screen prevalence in the full corpus and within-screen overlap with the broad PA/body/lifestyle screen.

The overlapping emotion screens were frequent (Table 4, Figure 4). Anxiety/worry matched 145,558 exchanges (69.41%), stress/overload 122,793 (58.55%), and depression/sadness 107,722 (51.37%). The within-emotion share that also met the broad PA screen was highest for sleep disturbance (69.48%), followed by shame/low self-worth (50.95%) and stress/overload (50.28%). Because an exchange could match several emotion categories, these figures are not a partition and should not be interpreted as prevalence estimates.

### 4.5. Physical Activity Keyword Patterns

**Figure 5:**
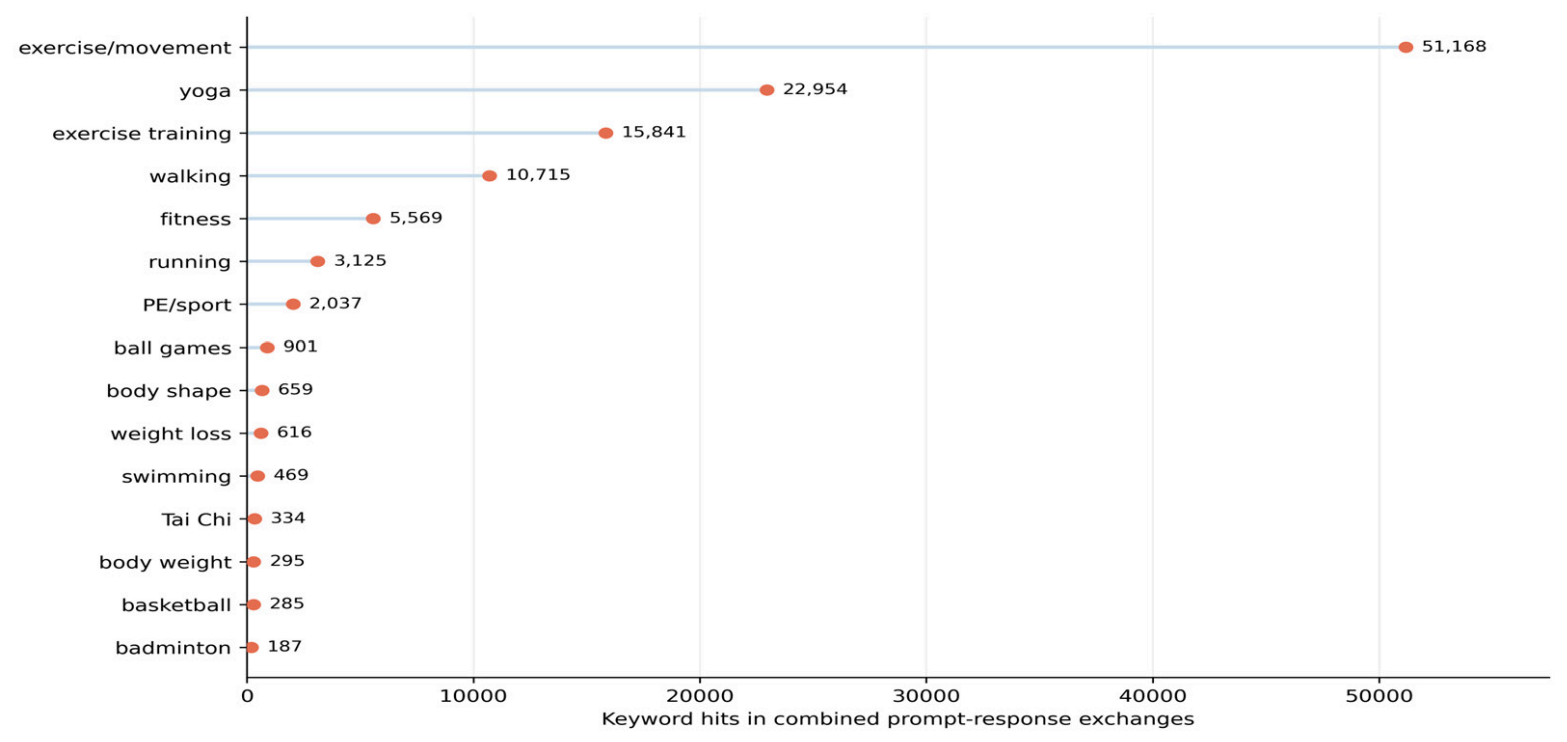
Most frequent PA-related keyword hits in combined prompt–response exchanges.

Keyword counts provided a descriptive check on the topic interpretation (Figure 5). The most frequent PA-related terms in combined exchanges were exercise/movement (51,168 hits), yoga (22,954), exercise training (15,841), walking (10,715), fitness (5569), running (3125), and PE/sport (2037). Lower-frequency terms included swimming, Tai Chi, basketball, and badminton. Counts reflect mentions rather than unique people, behaviours, recommendations, or intervention uptake, and the pooled unit prevents attribution to the prompt or response.

### 4.6. Sample-Based Movement-Related Expression Specificity and Scene Structure

Table 5 reports the narrower sample-based validation. Across 10,000 systematically sampled exchanges, 13.11% matched at least one explicit movement-related expression. Within Topic T4 (emotional outburst and relaxation regulation), 51.09% of assigned exchanges matched that screen. This narrower rate differs from the broad corpus-level PA/body/lifestyle screen because it excludes general body and lifestyle references. It remains an exchange-level textual indicator rather than evidence that an intervention was delivered or requested.

**Figure 6:**
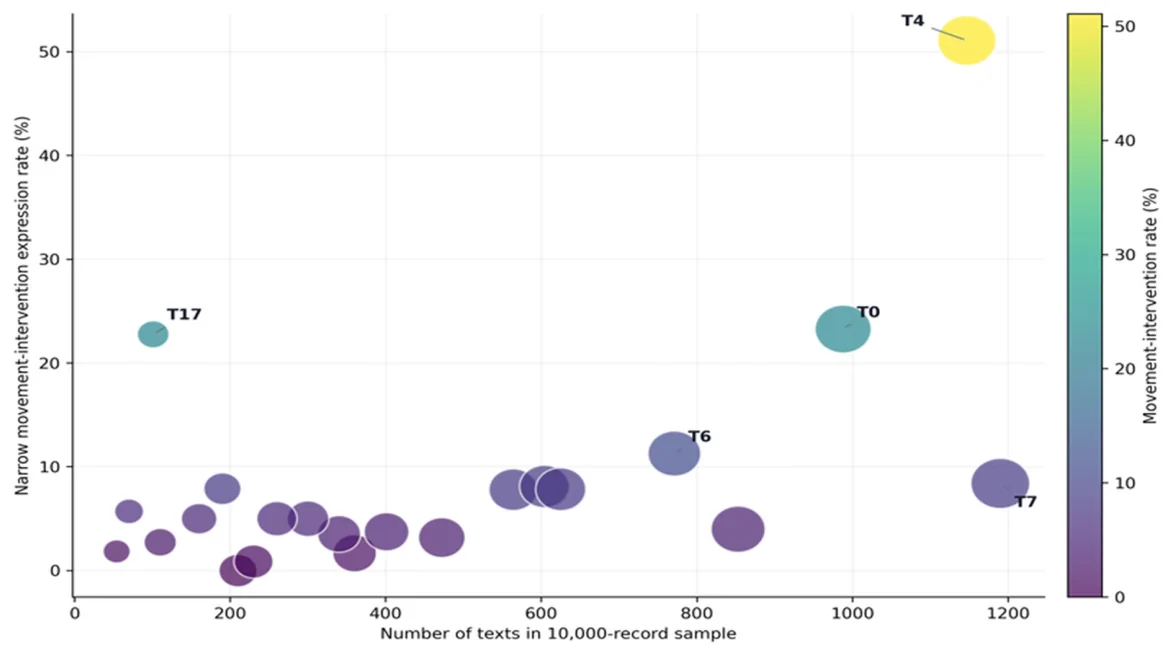
Sample-topic size and narrow movement-related expression rate.

Figure 6 shows that narrow movement-related expression rates did not increase monotonically with topic size. T4 combined a relatively large topic size with the highest screen rate, whereas smaller topics such as T17 also showed elevated rates. These are descriptive model-output patterns; they do not demonstrate clinical appropriateness, effectiveness, or speaker-specific demand.

### 4.7. Topic–Scenario Mapping

**Figure 7:**
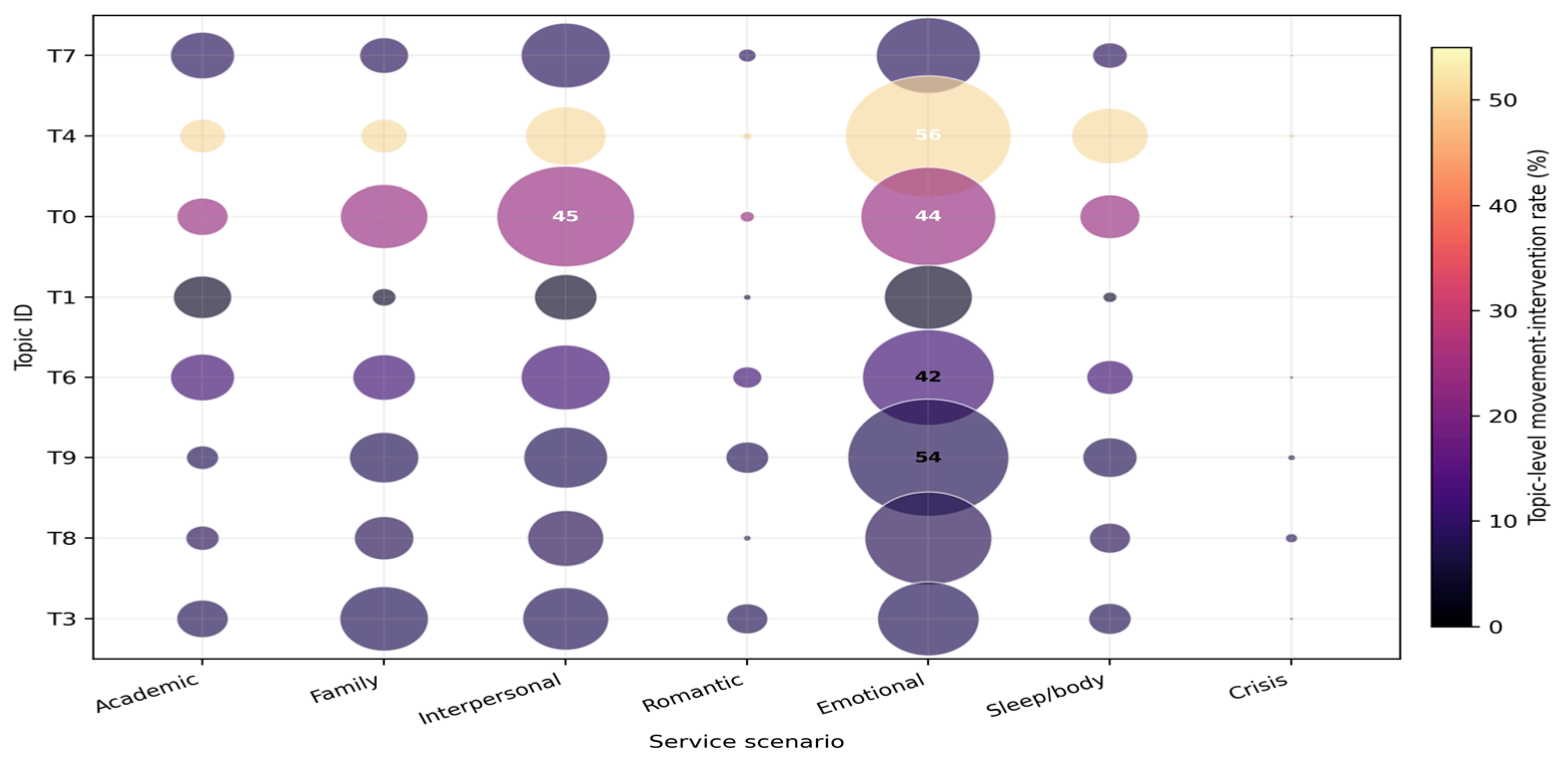
Scenario-screen strength and narrow movement-related expression fit in the 10,000-exchange validation sample.

**Figure 8:**
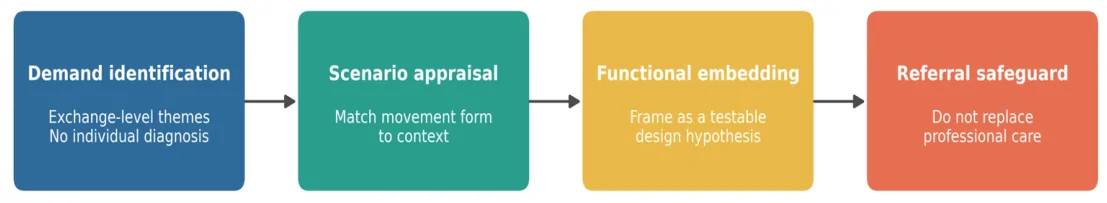
Hypothesis-generating framework for future evaluation of context-sensitive movement support.

The multi-label scene mapping placed movement-relevant sample topics most often alongside emotional-distress, sleep/psychosomatic, and interpersonal-relation screens (Table 6; Figure 7 and Figure 8). Within T4, 56.0% of exchanges matched the emotional-distress screen and 21.5% matched the sleep/psychosomatic screen; T9 showed a 54.1% emotional-distress match. T0 matched interpersonal-relation (44.6%) and emotional-distress (43.5%) screens. Crisis–risk matches were ≤2.3% across the displayed topics. These co-occurrences support scenario-specific hypotheses but do not establish referral effectiveness or a movement-related support pathway.

## 5. Discussion

This exploratory study used BERTopic, overlapping dictionary screens, and sample-based mapping to describe PA and psychosocial patterns in a large corpus of university student–AI mental health exchanges. Broad PA/body/lifestyle expressions appeared in more than one third of pooled exchanges, whereas explicit movement-related expressions were more selective (13.11% in the validation sample). The contrast underscores why general body or lifestyle references should not be interpreted as explicit movement advice, PA behaviour, or the delivery or uptake of any movement-based intervention.

The results generate a practical design hypothesis: generic “exercise more” language may be insufficient when a support exchange also contains emotional distress, body-image concerns, perceived incompetence, or social pressure. Future intervention research could test whether activity choice, graded goals, emotionally safe tasks, and relational support improve acceptability. The present descriptive analysis does not establish that these strategies are effective or appropriate for a particular student or clinical presentation. The scenario mapping further suggests a provisional sequence of demand screening, contextual appraisal, functional embedding of movement, and referral safeguards. This sequence is offered as a design framework for future evaluation, not as an empirically validated service pathway.

### 5.1. Implications for Sustainable Physical Education

The following implications are hypotheses for future PE and digital mental health design rather than evidence-based prescriptions. First, researchers could test whether treating emotional regulation as an explicit PE learning outcome improves engagement among students reporting distress. Low-threshold activities such as walking, stretching, yoga, Tai Chi, Baduanjin, and light aerobic exercise are candidate formats whose acceptability and safety require prospective evaluation. Second, future studies could compare autonomy-supportive options such as activity choice, micro-goals, peer companionship, and progress-focused feedback with standard delivery. The corpus does not establish which option works, for whom, or under what conditions.

Third, non-competitive and differentiated tasks may be worth testing for students who report low physical confidence, body-image concerns, or fear of public failure. Such adaptations should be evaluated for feasibility, acceptability, and unintended effects rather than assumed to be beneficial. Fourth, any movement-based support for psychologically distressed students should be situated within established safeguarding and referral procedures. Movement should not replace professional assessment or crisis care, particularly when self-harm or suicide-related content is present.

### 5.2. Limitations

Several limitations materially constrain interpretation. First, the archived record set does not document the upstream quality-screening criteria applied before the research team received the corpus, so the exact inclusion/exclusion rules used at the source platform cannot be fully reconstructed; this is a documentation gap in upstream processing, not uncertainty about the corpus’s institutional origin or authenticity, both of which are established in Section 3.2. Second, individual-level demographic variables exact age, sex, and year of study were not available at the point of data receipt, as were not focus of study. The population itself is known to be enrolled university students rather than an unverified general population, so the corpus is characterised consistently as adult university-student discourse; the student/school/family-context lexicon (student-, school-, parent-, and family-related terms) is used in the Results to describe topical references to school and family context, not to establish age. Third, prompts and responses were concatenated before modelling; although the origin of each field was known at the point of collection, the pooled analytical output prevents attribution of PA language, barriers, or suggestions to the student versus the AI system for any specific exchange.

Fourth, broad PA/body/lifestyle, emotion, and scenario screens are overlapping lexical rules and do not establish behaviour, diagnosis, or intervention delivery. Fifth, although the retrospective independent reassessment quantified reproducibility of the interpretive layer, its manual was frozen for the reassessment after the original exploratory analysis and therefore should not be interpreted as a preregistration. Agreement statistics assess reliability, not construct validity or direct measurement of SDT needs. Sixth, although the corpus-level BERTopic analysis was rerun using a fully specified and version-controlled pipeline and assessed across three random seeds, stability does not establish substantive or construct validity. The resulting topics remain dependent on the selected embedding model, dimensionality-reduction settings, clustering parameters, topic-reduction procedure, and interpretive labelling decisions. Seventh, the study did not evaluate the quality, safety, or clinical appropriateness of AI-generated responses and used no behavioural or intervention outcomes. Future work should use verified individual-level demographic metadata, separate prompt- and response-level models, preregistered dictionaries, version-controlled code and parameters for the topic model, topic stability tests, prospectively frozen coding manuals, external-coder replication, and validation against behavioural and clinical measures.

## 6. Conclusions

This exploratory study analysed 209,715 paired university student–AI mental health exchanges using a BERTopic-based framework. Eighteen corpus-level topics and seven overlapping PA-related thematic screens were retained. In a systematic 10,000-exchange validation sample, 13.11% of exchanges matched narrow movement-related expressions, including 51.09% of exchanges assigned to the emotional-outburst and relaxation–regulation topic. The results describe how PA-related language co-occurred with emotional, university, family, interpersonal, sleep, and body-related text in combined prompt–response exchanges.

## Figures and Tables

**Table 1:** Corpus summary and analytical scope.

Indicator	Value
Total Q&A records (corpus-level denominator)	209,715
Filtered sampling frame used only to draw the complementary 10,000-exchange sample	178,062
Student/school/family-context lexicon matches (topical screen, not age-verifying)	83,215 (39.68%)
Broad PA/body/lifestyle screen matches	82,464 (39.32%)
Overlap of context and broad PA/body/lifestyle screens	35,141 (16.76% of all records; 42.61% of PA-related subset)
Retained corpus-level themes	18
Retained physical activity-related subthemes	7
Systematic exchange sample for complementary scenario analysis	10,000 records
Narrow movement-related expression rate in sample	13.11%

**Table 2:** Ten largest of 18 corpus-level topics and overlap with the broad PA/body/lifestyle screen.

Corpus-Level Topic-Label	Assigned Exchanges	All (%)	Broad PA-Screen Positive	Within-Topic PA Rate (%)	Representative Terms
Anxiety, worry and fear	61,750	29.44	29,098	47.12	anxiety; worry; fear; tension; panic; uncertainty
Academic pressure and school adaptation	47,923	22.85	23,707	49.47	study; school; exam; grade; teacher; classmates
Family caregiving and parent–child conflict	46,776	22.30	15,964	34.13	parents; child; mother; father; family; conflict
Depressive mood and hopelessness	18,707	8.92	6023	32.20	depression; sadness; low mood; despair; pain
Romantic relationship and breakup distress	17,354	8.28	4212	24.27	relationship; breakup; partner; love; loss
Interpersonal conflict and social anxiety	6003	2.86	1597	26.60	friend; social; roommate; communication; rejection
Marriage and intimate-partner conflict	2501	1.19	506	20.23	marriage; spouse; intimacy; conflict
Self-worth, inferiority and identity concerns	2316	1.10	417	18.01	inferiority; self-worth; shame; identity
Workplace stress and career confusion	1401	0.67	268	19.13	work; career; boss; resignation; confusion
Other/general emotional consultation	1198	0.57	9	0.75	distress; hesitation; uncertainty; advice

**Table 3:** Overlapping PA-related thematic screens.

Overlapping PA-Related Thematic Screen	Matched Exchanges	All Exchanges (%)	Broad PA-Screen-Positive Exchanges (%)
Co-occurrence of movement-related and emotional-regulation expressions	79,452	37.89	96.35
Health habit and lifestyle management	49,507	23.61	60.03
Social and peer support in activity	49,433	23.57	59.94
Motivation and persistence barriers	32,340	15.42	39.22
Exercise overuse or pressure	5843	2.79	7.09
PE/school sport anxiety	5353	2.55	6.49
Body image and weight concern	1475	0.70	1.79

**Table 4:** Overlapping emotion screens and broad PA-screen overlap.

Overlapping Emotion Screen	Matched Exchanges	All Exchanges (%)	Broad PA-Screen Positive	PA Within Emotion Screen (%)
Anxiety and worry	145,558	69.41	66,056	45.38
Stress and overload	122,793	58.55	61,736	50.28
Depression and sadness	107,722	51.37	49,207	45.68
Loneliness and social isolation	71,613	34.15	34,726	48.49
Shame and low self-worth	58,190	27.75	29,647	50.95
Sleep disturbance	30,512	14.55	21,200	69.48
Anger and conflict	28,349	13.52	9567	33.75
Self-harm- and suicide-related language	5382	2.57	2300	42.74

**Table 5:** Sample topics with the highest narrow movement-related expression rates. The denominator for each rate is the number of sampled exchanges assigned to that topic.

Topic	Sample-Based Topic-Label	Narrow Movement-Related Expression Rate Within Topic (%)	Main Movement Expressions
T4	Emotional outburst and relaxation regulation	51.09	exercise (230), breathing (217), yoga (132), walking (76), training (56), running (23), fitness (19), relaxation training (19)
T0	Interpersonal relations and emotional entanglement	23.28	exercise (99), yoga (63), breathing (56), training (29), walking (23), running (9), fitness (9)
T17	Depressive crisis and risk support	22.77	exercise (14), training (7), breathing (3), yoga (2), PE (1), relaxation training (1)
T6	Anxiety, stress and emotion regulation	11.28	exercise (22), yoga (13), breathing (9), walking (6), relaxation training (4), running (3), fitness (3)

**Table 6:** Multi-label scenario association rates in the 10,000-exchange validation sample.

Sample Topic	Academic Pressure	Family/Parent–Child	Interpersonal Relations	Romantic Relations	Emotional Distress	Sleep/ Psychosomatic	Crisis Risk
T7	17.4%	12.5%	26.1%	3.6%	31.6%	8.2%	0.3%
T4	11.5%	11.6%	23.0%	1.7%	56.0%	21.5%	0.9%
T0	13.2%	25.5%	44.6%	2.8%	43.5%	16.1%	0.6%
T1	15.5%	5.2%	16.9%	1.3%	25.7%	2.7%	0.0%
T6	17.4%	16.9%	26.1%	6.6%	42.2%	11.7%	0.6%
T9	7.5%	19.2%	24.2%	10.6%	54.1%	14.2%	1.3%
T8	7.8%	15.9%	21.5%	1.3%	40.4%	10.1%	2.3%
T3	13.3%	25.9%	25.0%	10.1%	30.7%	10.5%	0.5%

## Data Availability

The raw corpus is not publicly available because it contains sensitive adult mental-health text and residual deductive-disclosure risk, including self-harm-related language. The accompanying attachment package provides the coding manual, separate coder ratings, adjudication log, reliability calculations and script, aggregated outputs, dictionaries, configuration files, and non-text document topic identifiers. Access to any additional non-public analytical material remains subject to ethics approval, institutional data-governance restrictions, and protection of third-party rights.

## Supplementary Materials

The following supporting information can be downloaded at: https://www.mdpi.com/article/10.3390/healthcare14162547/s1. The accompanying attachment Package contains the frozen topic-label and SDT-mapping coding manual, topic codebook, separate locked pre-adjudication records for Coder A and Coder B, adjudication log, reliability summary, executable reliability-analysis script, dictionary resources, and computational configuration files.
